# Auxin, Abscisic Acid and Jasmonate Are the Central Players in Rice Sheath Rot Caused by *Sarocladium oryzae* and *Pseudomonas fuscovaginae*

**DOI:** 10.1186/s12284-020-00438-9

**Published:** 2020-11-26

**Authors:** K. J. Peeters, M. Ameye, K. Demeestere, K. Audenaert, M. Höfte

**Affiliations:** 1 Department of Plants and Crops, Laboratory of Phytopathology, Faculty of Bioscience Engineering, Ghent University, Coupure Links 653, B-9000 Ghent, Belgium; 2 Laboratory of Applied Mycology and Phenomics, Department of Plants and Crops, Faculty of Bioscience Engineering, Ghent University, Valentin Vaerwyckweg 1, B-9000 Ghent, Belgium; 3 Department of Green Chemistry and Technology, Research Group EnVOC, Ghent University, Coupure Links 653, B-9000 Ghent, Belgium

**Keywords:** Abscisic acid, Auxin, H^+^-ATPase, Jasmonate, *Pseudomonas fuscovaginae*, Rice, *Sarocladium oryzae*, Sheath rot, Helvolic acid, Fuscopeptin

## Abstract

Sheath rot is an emerging rice disease that causes severe yield losses worldwide. The main causal agents are the toxin producers *Sarocladium oryzae* and *Pseudomonas fuscovaginae*. The fungus *S. oryzae* produces helvolic acid and cerulenin and the bacterium *P. fuscovaginae* produces cyclic lipopeptides. Helvolic acid and the lipopeptide, fuscopeptin, inhibit membrane-bound H^+^-ATPase pumps in the rice plant. To manage rice sheath rot, a better understanding of the host response and virulence strategies of the pathogens is required. This study investigated the interaction of the sheath rot pathogens with their host and the role of their toxins herein. *Japonica* rice was inoculated with high- and low-helvolic acid-producing *S. oryzae* isolates or with *P. fuscovaginae* wild type and fuscopeptin mutant strains. During infection, cerulenin, helvolic acid and the phytohormones abscisic acid, jasmonate, auxin and salicylic acid were quantified in the sheath. In addition, disease severity and grain yield parameters were assessed. Rice plants responded to high-toxin-producing *S. oryzae* and *P. fuscovaginae* strains with an increase in abscisic acid, jasmonate and auxin levels. We conclude that, for both pathogens, toxins play a core role during sheath rot infection. *S. oryzae* and *P. fuscovaginae* interact with their host in a similar way. This may explain why both sheath rot pathogens cause very similar symptoms despite their different nature.

**Supplementary Information:**

The online version contains supplementary material available at 10.1186/s12284-020-00438-9.

## Background

Rice sheath rot is an emerging disease that affects all rice growing areas worldwide. Different pathogens have been associated with this disease and causal agents cannot be distinguished based on symptoms (Cottyn et al. 1996; Bigirimana et al. 2015). High-yielding commercial rice varieties are very susceptible to rice sheath rot. Furthermore, breeding for resistant varieties is difficult because there are various sheath rot pathogens and there is a lack of knowledge about their infection strategies (Bigirimana 2016; Sakthivel 2001; Ayyadurai et al. 2005; Chauhan et al. 2017a; Mvuyekure et al. 2017). The main pathogens associated with this disease are the fungus *Sarocladium oryzae* and the bacterium *Pseudomonas fuscovaginae*. Also different *Fusarium* spp., such as *Fusarium andiyazi*, *F. proliferatum*, *F. verticillioides* and *F. fujikuroi* can cause sheath rot symptoms (Bigirimana 2016; Wulff et al. 2010). Both *S. oryzae* and *P. fuscovaginae* are seed-borne which could explain the fast spreading of these pathogens (Batoko et al. 1997; Ayyadurai et al. 2005; Adorada et al. 2015). All sheath rot pathogens cause similar greyish-brown necrotic lesions on the uppermost leaf sheath that encloses the youngest panicle. Depending on the severity of the infection, diseased plants produce brown, sterile or empty seeds or form no panicle at all (Bigirimana et al. 2015; Weeraratne et al. 2020).

The fungus *S. oryzae* was first described as *Acrocylindrium oryzae* in 1922 in Taiwan (Sawada 1922) and has spread to at least 36 countries (CABI, 2020). Yield losses range from 20% up to 85% and are the highest in hot and humid conditions (Sakthivel 2001; Panda and Mishra 2019). Cell wall degrading enzymes and the toxins cerulenin and helvolic acid are the main virulence factors (Ayyadurai et al. 2005; Nandakumar et al. 2007). Cerulenin, a hexaketide amide, inhibits fatty acid and polyketide biosynthesis in other fungi and in plants (Omura 1976; Wenzel et al. 2011). The tetracyclic triterpenoid helvolic acid causes chlorosis on *Poaceae* (Tschen et al. 1997; Sakthivel et al. 2002). It captures free magnesium ions (Mg^2+^) which are needed in chlorophyll biosynthesis, photosynthesis and carbohydrate transport (Tschen et al. 1997; Sakthivel et al. 2002; Cakmak and Kirkby 2008; Farhat et al. 2016). We have shown before that the *S. oryzae* population is very diverse in its toxigenicity and virulence with the most pathogenic isolates producing the highest amounts of helvolic acid in the rice sheath. These virulent isolates were closely related to each other and were found to be phenotypically stable. The less virulent isolates, on the contrary, formed sectors in stressed conditions. Sectorization is a sign of phenotypic instability and was found to affect helvolic acid production (Peeters et al. 2020).

The Gram-negative bacterium *P. fuscovaginae* causes sheath brown rot in rice plants. It was first reported in 1976 in Japan and is able to cause a total yield loss (Tanii et al. 1976; Razak et al. 2009; Weeraratne et al. 2020). Sheath brown rot has been reported in 35 countries (CABI, 2020) and is mostly associated with cold and tropical highlands (Batoko et al. 1997; Bigirimana 2016). The phytotoxic cyclic lipopeptides (also called lipodepsipeptides) syringotoxin, fuscopeptin A and fuscopeptin B are involved in the disease development of *P. fuscovaginae* (Flamand et al. 1996; Batoko et al. 1997). Syringotoxin is a cyclic lipopeptide with 9 amino acid residues that is also produced by *P. syringae pv. syringae* pathogenic on citrus trees (Ballio et al. 1990; Flamand et al. 1996). Fuscopeptin A and B contain 19 amino acid residues and are structurally very similar to the thoroughly studied *P. syringae* toxin, syringopeptin (Coraiola et al. 2008). The three toxins are produced concomitantly and work synergistically. A toxin mixture of syringotoxin, fuscopeptin A and B triggers necrosis on the rice sheath and, since *P. fuscovaginae* is pathogenic to all *Poaceae*, they are considered non-host-specific (Miyajima et al. 1983; Batoko et al. 1998). Due to their amphiphilic nature, the toxins act as surfactant and interact with biological membranes thereby forming pores which cause leakage of protons, disrupting the proton gradient (Batoko et al. 1998; Coraiola et al. 2008; Patel et al. 2014). Just like helvolic acid, fuscopeptin A and B inhibit H^+^-ATPases. They interact directly with the proton pump and inactivate the catalytic centre (Batoko et al. 1998). Moreover, they show antifungal activity, but not against *S. oryzae* (Ballio et al. 1996).

Pathogens often use toxins to suppress pathways that confer resistance or to manipulate essential developmental or physiological processes. With this, they aim to facilitate host entry, colonization or feeding. Because of their important role as signalling molecules in the fine-tuning of biotic and abiotic stress responses, phytohormones are often manipulated by pathogens (De Vleesschauwer et al. 2013; Yang et al. 2013). Pathogens typically target the archetypal jasmonate (JA), salicylic acid (SA) and ethylene (ET) dependent defence hormone pathways, which are important in the immune response of rice (De Vleesschauwer et al. 2013; Yang et al. 2013; Patkar and Naqvi 2017). Also the biosynthesis and signalling of abscisic acid (ABA), and the growth hormones cytokinin and auxin (AUX) are often affected (Kazan and Lyons 2014; Ma and Ma 2016; Patkar and Naqvi 2017). ABA is widely studied for its role in tolerance to abiotic stress, such as salinity, drought and cold. However, ABA also fulfils a role in plant immunity either alone or through a complicated network of antagonistic and synergistic interactions with other hormone signalling pathways (De Vleesschauwer et al. 2010; Yang et al. 2013; Ku et al. 2018). The main AUX in rice is indole acetic acid (IAA). Its role in plant defence is mostly antagonistic. Therefore, pathogens often increase IAA levels during infection by production and secretion of IAA or by stimulation of the hosts IAA biosynthesis (De Vleesschauwer et al. 2013; Yang et al. 2013).

Sheath rot pathogens cause nearly identical symptoms on the sheath and the panicle. Since there is a partial overlap in the mode of action of their toxins, we hypothesize that these toxins play a crucial role in virulence and that both pathogens elicit similar phytohormone responses (Batoko et al. 1997, 1998; Coraiola et al. 2008; Hoagland 2009). Previous research has shown the importance of fuscopeptin (Patel et al. 2014; Weeraratne et al. 2020) and helvolic acid (Peeters et al. 2020) in symptom development by resp. *P. fuscovaginae* and *S. oryzae*. Their mode of action has been thoroughly studied in vitro but the role in the infection process and their production profile needs further investigation (Batoko et al. 1998; Coraiola et al. 2008; Hoagland 2009). For adequate disease management, it is of crucial importance that we understand the virulence strategies of the sheath rot pathogens and the host defence response (De Vleesschauwer et al. 2013). In previous research, foliar application of SA did not reduce disease severity or decrease the yield losses caused by *S. oryzae* infection (Chauhan et al. 2017b). Rice plants overexpressing WRKY13 were more resistant to *S. oryzae* (Lilly and Subramanian 2019). This transcription factor (TF) plays an important role in the JA-SA crosstalk and in the crosstalk between biotic and abiotic stress (Qiu et al. 2007; Xiao et al. 2013; De Vleesschauwer et al. 2014). A balanced nutrient status and soil application of magnesium, copper, potassium and calcium could also reduce disease incidence caused by *S. oryzae* (Tschen et al. 1997; Laha et al. 2016; Zhang et al. 2019). While studies on the host immune response to *S. oryzae* are very limited, information about the host response and resistance factors against *P. fuscovaginae* is completely lacking.

Here, we explore the role of the toxins cerulenin, helvolic acid and fuscopeptin in the interaction of *S. oryzae* and *P. fuscovaginae* with rice. *S. oryzae* isolates that differ in their toxin production in vitro and *in planta* (Peeters et al. 2020) were used to investigate how these compounds influence symptom development, yield losses and the hormone balance of the rice plant. For *P. fuscovaginae*, a wild type strain was compared with its fuscopeptin mutant (Patel et al. 2014; Weeraratne et al. 2020). At different infection stages, toxin and phytohormone levels were measured to better understand the virulence strategies of the sheath rot pathogens.

## Results

### Disease Assessment, Phytohormone and Toxin Levels during *Sarocladium oryzae* Infection

**Fig. 1:**
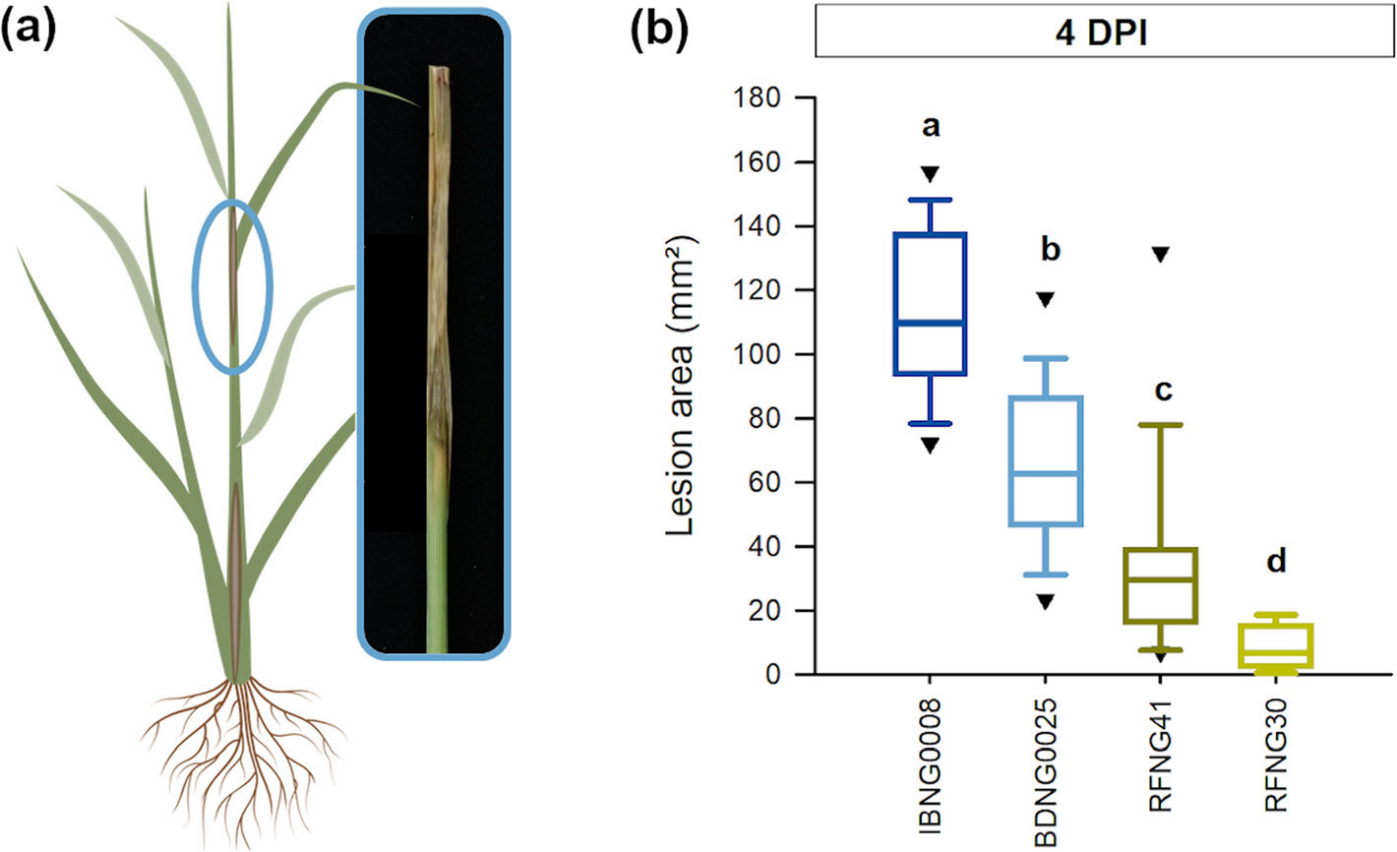
Virulence of Sarocladium oryzae on Kitaake rice plants. When 7 weeks old, rice plants were inoculated in the axil of the second youngest leaf by use of the standard grain inoculum technique (**a**). Disease was evaluated at 4 days post inoculation (DPI) with *S. oryzae* isolates IBNG0008 (dark blue), BDNG0025 (light blue), RFNG41 (dark green) and RFNG30 (light green) (**b**). All boxplots show the median with the first and third quartile, the whiskers show the minimum and maximum values. Outliers and extreme values are represented by triangles. Boxplots marked with different letters are statistically different (ANOVA, *n* = 20, α = 0.05)

To study the interaction of *S. oryzae* with its host and the role of its toxins cerulenin and helvolic acid herein, rice plants were inoculated with four isolates (Table 1) that were earlier shown to differ in virulence and toxin production (Table S1; Peeters et al. 2020). The rice sheaths of the second youngest leaf, enclosing the colonized grain, were collected at 4 h after inoculation (0 days post inoculation ((DPI)) and 2 and 4 DPI. Although all isolates were able to cause long, brown necrotic lesions on the sheath enclosing the young panicle, significant differences were observed in the lesion size at 4 DPI (Fig. 1).

**Table 1:** Characteristics of the *Sarocladium* isolates used in this study

		Phylogenic group/species	
***S. oryzae*** isolate	Origin	Peeters et al. 2020^a^	Ou et al. 2020^b^
IBNG 0008	Rice sheaths, Nigeria	Group 3	*S. sparsum*
IBNG 0009	Rice sheaths, Nigeria	Group 3	*S. sparsum*
BDNG 0025	Rice sheaths, Nigeria	Group 3	*S. sparsum*
RFRG 2	Rice sheaths, Rwanda	Group 1	*S. oryzae*
CBS 180.74	Rice, India	Group 1	*S. oryzae*
RFNG 30	Rice sheaths, Rwanda	Group 2	*S. attenuatum*
RFNG 122	Rice sheaths, Rwanda	Group 2	*S. attenuatum*
RFBG 3	Rice sheaths, Rwanda	Group 2	*S. attenuatum*
RFNG 41	Rice sheaths, Rwanda	Group 2	*S. attenuatum*
BDNG 0005	Rice sheaths, Nigeria	Group 2	*S. attenuatum*

^a^Maximum-likelihood analysis based on ACT and ITS, ^b^ Based on Maximum-likelihood analysis with on ACT, ITS, LSU and TUB2 Ou et al. (2020) have proposed to reclassify *S. oryzae* in three species, which correspond with the 3 groups that were described by Peeters et al. (2020)

**Fig. 2:**
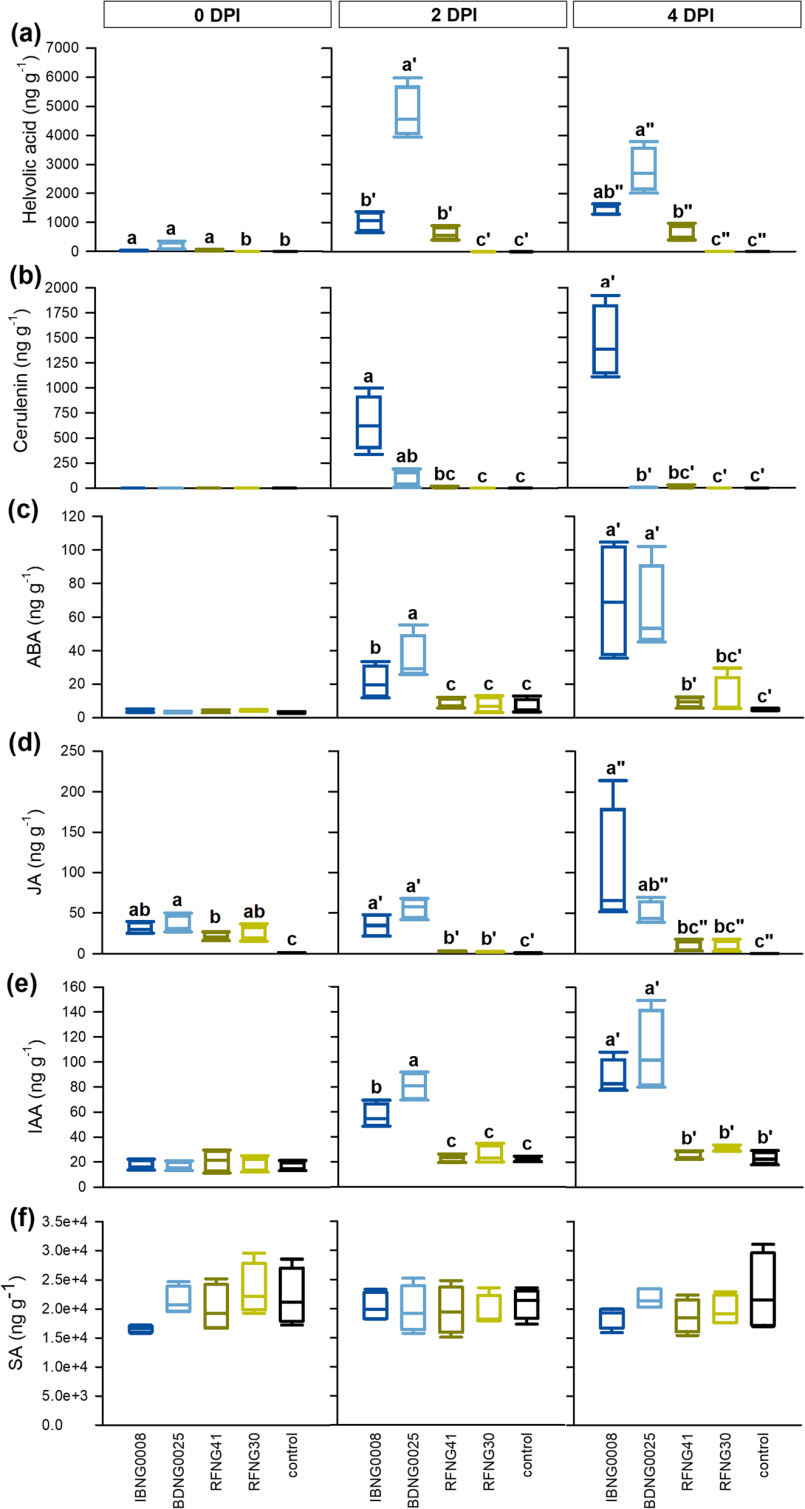
Phytohormone and toxin levels in the rice sheath during *Sarocladium oryzae* infection. When 7 weeks old, Kitaake rice plants were inoculated with *S. oryzae* using the standard grain inoculum technique. The levels of helvolic acid (**a**), cerulenin (**b**), abscisic acid (**c**), jasmonate (**d**), auxin (**e**) and salicylic acid (**f**) were measured in sheath samples collected after 4 h (0 days post inoculation (DPI)) and at 2 DPI and 4 DPI. The four *S. oryzae* isolates are represented by dark blue (IBNG0008), light blue (BDNG0025), dark green (RFNG41) and light green (RFNG30) boxplots and black boxplots show the results of healthy control plants. All boxplots represent the median with the first and third quartile, the whiskers show the minimum and maximum values. Boxplots marked with different letters are statistically different (ANOVA, Dunn’s or Mood’s Median test, *n* = 4, α = 0.05)

At 4 h after infection, low amounts of helvolic acid were detected in rice sheaths inoculated with the three most pathogenic isolates IBNG0008, BDNG0025 and RFNG41 (Fig. 2a). During the first 48 h, their helvolic acid production increased. Isolate BDNG0025 showed the highest helvolic acid production at every time point. The least pathogenic isolate RFNG30, on the contrary, produced trace amounts of helvolic acid only at one replicate at 4 DPI, while further no helvolic acid could be measured at all (Fig. 2a). Cerulenin was not detected at 4 h after inoculation. At 2 and 4 DPI, high amounts of cerulenin were measured in sheaths infected with IBNG0008, while BDNG0025 and RFNG41 showed a lower production. At none of the stages of the infection process, cerulenin could be detected for the isolate with the lowest virulence (RFNG30) (Fig. 2b).

In addition to the toxins, the levels of the phytohormones ABA, JA, IAA and SA were measured (Fig. 2c-f). At 4 h after inoculation (0 DPI), no changes in ABA concentration could be observed. At 2 DPI, ABA levels were elevated in plants inoculated with the two most pathogenic isolates (IBNG0008 and BDNG0025) and, by 4 DPI, ABA levels were further increased (Fig. 2c). Compared to the healthy control plants, a ten-fold increase of ABA was measured for the virulent isolates (IBNG0008 and BDNG0025) while ABA levels were only doubled in plants inoculated with RFNG41 and RFNG30 (Fig. 2c). JA, on the other hand, showed a transient increase in response to all *S. oryzae* isolates. By 2 DPI, JA levels again decreased in sheaths infected by isolates with low pathogenic potential (RFNG41 and RFNG30) and by 4 DPI, JA levels were still comparable to the concentration of JA in the healthy control plants. For the virulent isolates IBNG0008 and BDNG0025, on the contrary, JA levels stayed elevated during the rest of the infection (Fig. 2d). IAA levels were altered by *S. oryzae* in a similar pattern as ABA. At 4 h after inoculation, IAA levels were equal in all treatments. By 2 DPI, the virulent isolates (IBNG0008 and BDNG0025) had caused an increase of IAA which stayed elevated. In less diseased plants, similar levels as in the healthy control plants were measured at all sampling points (Fig. 2e). SA levels did not change in response to *S. oryzae infection*, although a small, not significant decrease of SA was observed at 4 h after inoculation of IBNG0008 (Fig. 2f**)**.

**Fig. 3:**
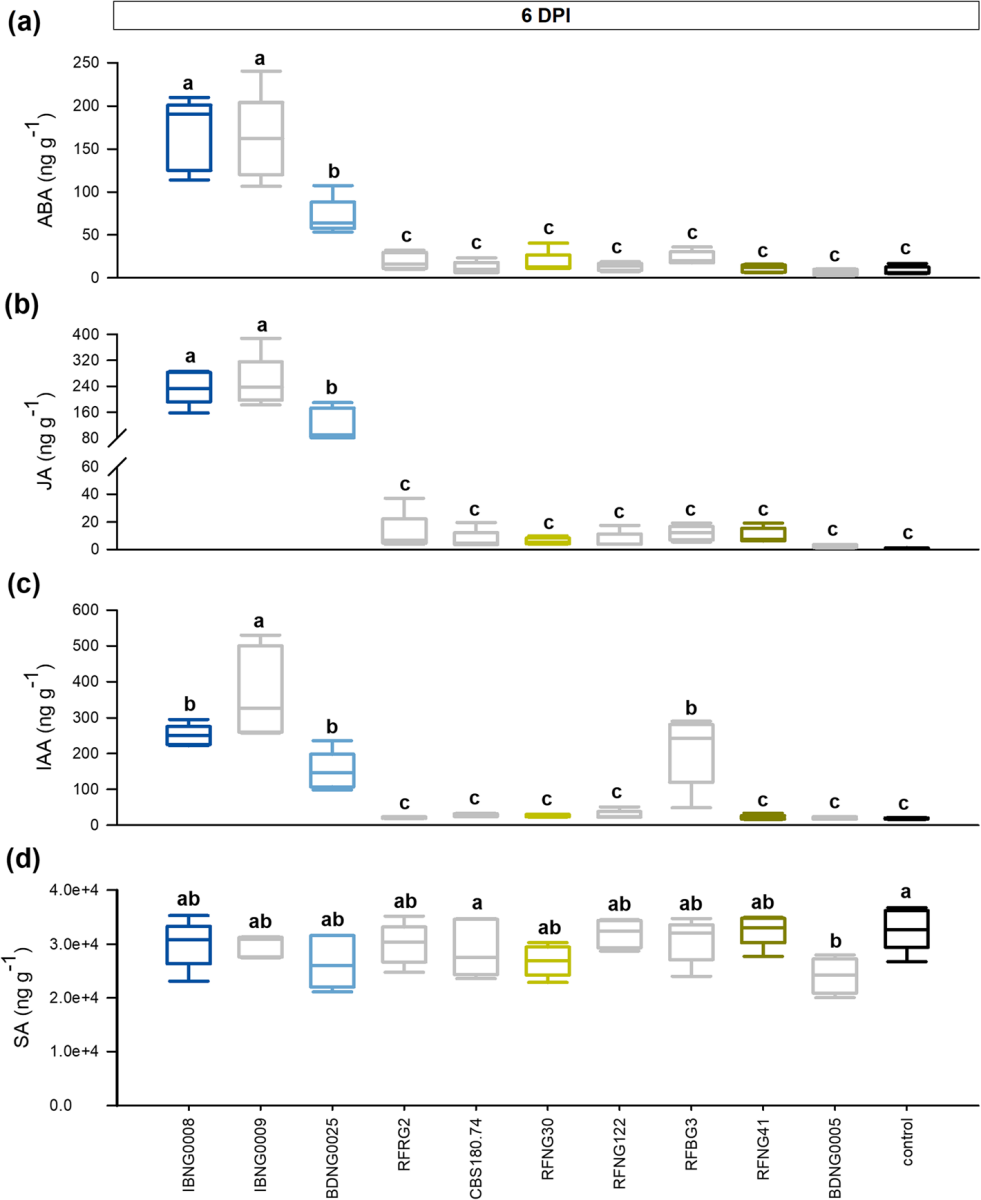
Phytohormone levels in sheath rot lesion caused by Sarocladium oryzae infection. When 7 weeks old, rice plants were inoculated with various *S. oryzae* isolates using the standard grain inoculum technique. Sheath samples were collected at 6 days post inoculation (DPI) and the levels of abscisic acid (**a**), jasmonate (**b**), auxin (**c**) and salicylic acid (**d**) were measured. The four *S. oryzae* isolates that were described above are represented by dark blue (IBNG0008), light blue (BDNG0025), dark green (RFNG41) and light green (RFNG30) boxplots; black boxplots show the results of the healthy control plants. All boxplots represent the median with the first and third quartile, the whiskers show the minimum and maximum values. Boxplots marked with different letters are statistically different (ANOVA, *n* = 5, α = 0.05)

To further elucidate the correlation between the hormonal response, the toxin production and the virulence, a wide selection of well characterized *S. oryzae* isolates was used (Table 1). Figure 3 shows the phytohormone levels in the rice sheaths of the second youngest leaf at 6 DPI. The corresponding virulence data and toxin levels measured in the sheaths have been reported in previously published work (Peeters et al. 2020) and the averages are listed in Table S1. In agreement with the results shown in Fig. 2, ABA, JA and IAA levels were the most elevated in rice sheaths infected with IBNG0008 and BDNG0025. Together with IBNG0009, to which phytohormone levels responded in a similar way, these isolates were the most pathogenic (Fig. 3a-c, Table S1). All three isolates produced high levels of helvolic acid, while their cerulenin production strongly differed. In agreement with Fig. 2, BDNG0025 produced significantly less cerulenin than IBNG0008 (Table S1). One isolate (BDNG0005) produced a similar amount of helvolic acid as BDNG0025 but did not trigger ABA, JA or IAA (Fig. 3a-c, Table S1). It did however cause a small but significant decrease of SA compared to the healthy control plants and this isolate was the least virulent of all (Peeters et al. 2020). The isolate RFBG3 elevated levels of IAA while it caused only minor symptoms and produced no cerulenin or helvolic acid (Fig. 3a-c, Table S1).

### Disease Assessment and Phytohormone Levels during *Pseudomonas fuscovaginae* Infection

**Fig. 4:**
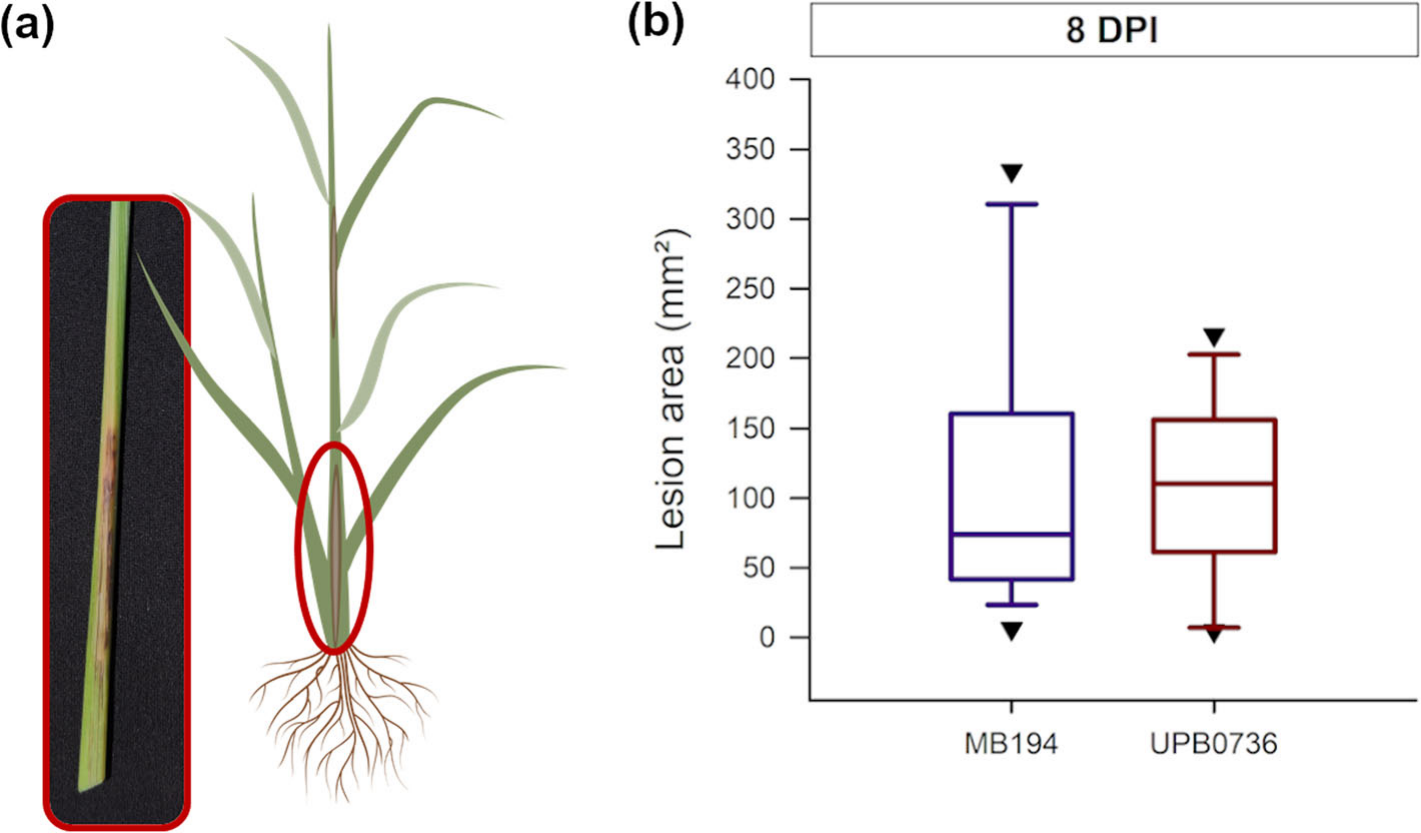
Virulence of Pseudomonas fuscovaginae wild type strains on Kitaake rice plants. When 7 weeks old, rice plants were inoculated with P. fuscovaginae. (**a**) Lesions on the rice sheath at 8 days post inoculation (DPI). Rice plants were inoculated with bacterial solution and scored at 8 DPI (**b**). The purple boxplot represents strain MB194 and the red boxplot represents UPB0736. All boxplots show the median with the first and third quartile, the whiskers show the minimum and maximum values. Outliers and extreme values are represented by triangles. The lesions did not differ significantly (Wilcoxon rank sum test, *n* = 25, α = 0.05)

Two wild type strains were used to study the phytohormone response to *P. fuscovaginae* infection. For this, rice plants were injected with a bacterial solution and disease was evaluated by measuring the lesion size around the inoculation point (Fig. 4). The strains were equally virulent and caused similar brown, necrotic lesions on both the sheath and the stem of the rice plant (Fig. 4a).

**Fig. 5:**
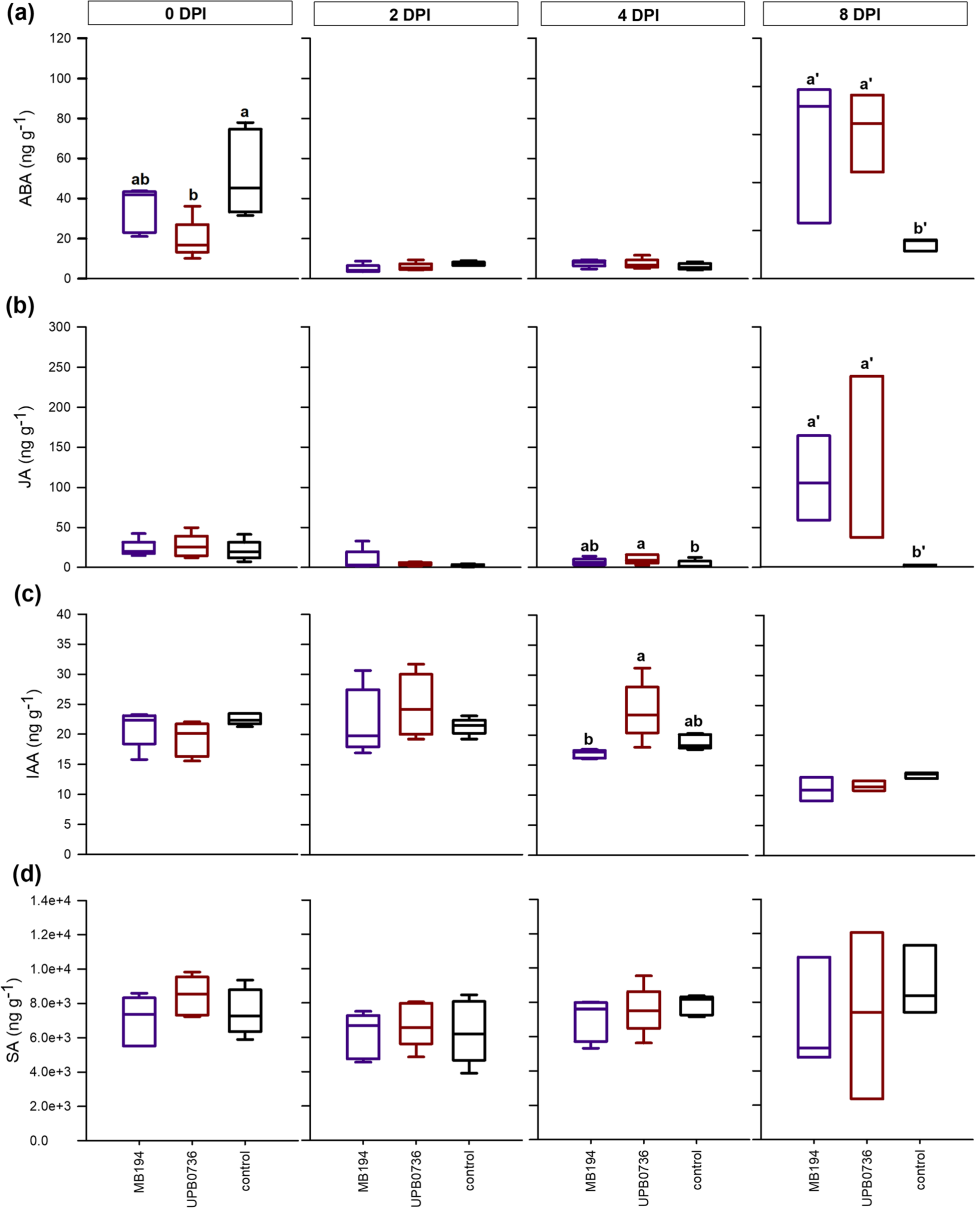
Phytohormone levels in the rice sheath during infection of Pseudomonas fuscovaginae wild type strains. When 7 weeks old, rice plants were inoculated with P. fuscovaginae by injecting a bacterial solution. The levels of abscisic acid (**a**), jasmonate (**b**) auxin (**c**) and salicylic acid (**d**) were measured after 4 h (0 days post inoculation (DPI)) and at 2 DPI, 4 DPI and 8 DPI. Purple boxplots represent strain MB194 and red boxplots represent strain UPB0736. Black boxplots show the results of the healthy control. All boxplots represent the median with the first and third quartile, the whiskers show the minimum and maximum values. Boxplots marked with different letters are significantly different (ANOVA or Dunn’s test, *n* = 5, α = 0.05)

At 4 h after inoculation (0 DPI) and 2, 4 and 8 DPI, samples of the rice sheath around the junction point were collected. Figure 5 shows that the rice plants strongly responded to the inoculation method by elevating ABA. The strongest increase was observed in the control plants which were inoculated with saline solution (Fig. 5a). By 2 DPI, ABA levels had decreased in all conditions. A late response to both wild type strains was observed at 8 DPI for both ABA and JA (Fig. 5a,b). IAA, on the other hand, showed a small transient increase in response to *P. fuscovaginae*. At 2 DPI, the IAA increase had started and by 4 DPI, IAA levels had dropped again for MB194 while in UPB0736 infected plants, IAA was still elevated. By 8 DPI, IAA concentrations were equal to the healthy control plants (Fig. 5c). SA did not respond to *P. fuscovaginae* infection (Fig. 5d).

**Fig. 6:**
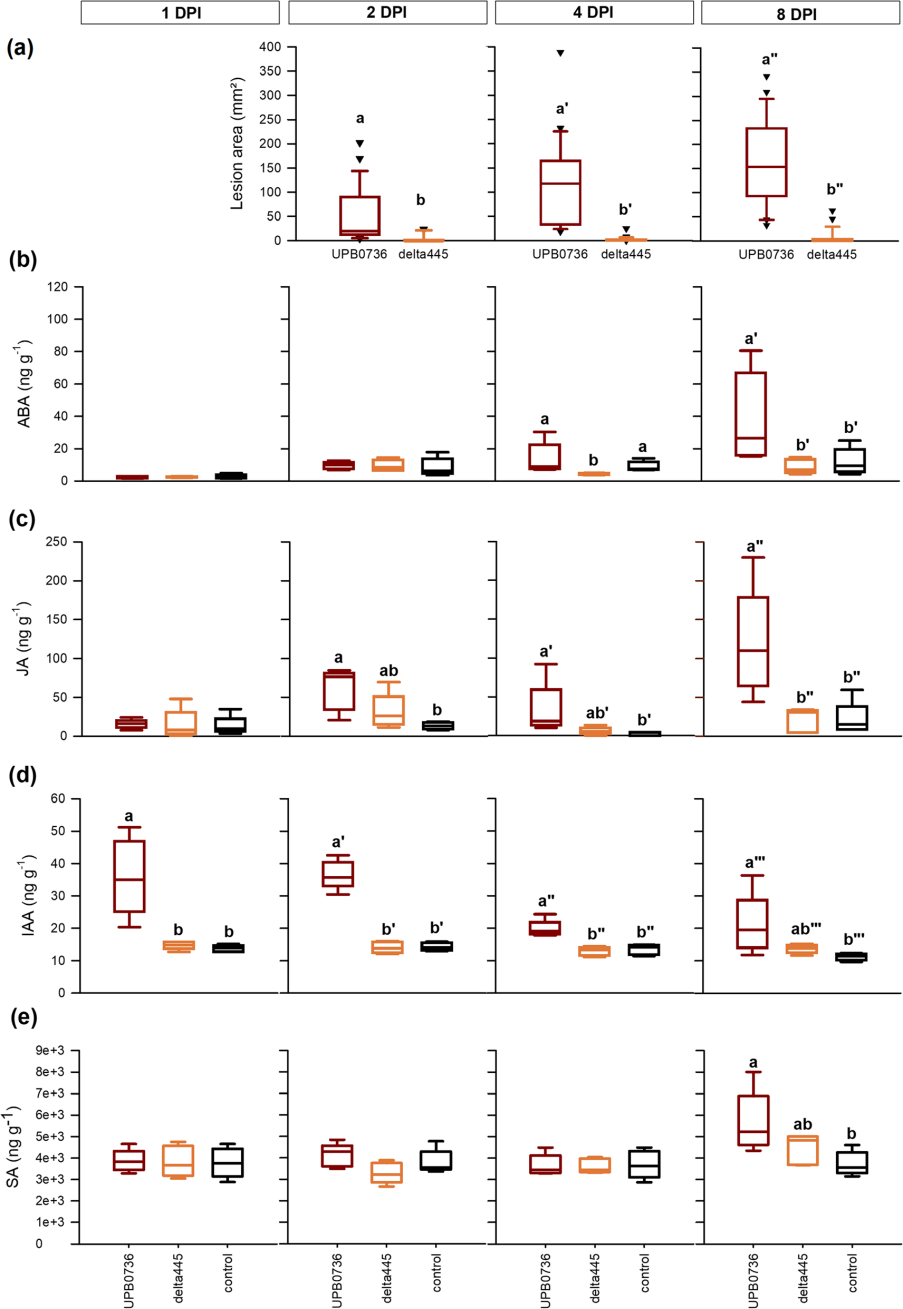
Comparison of the Pseudomonas fuscovaginae wild type strain UPB0736 (red) and its fuscopeptin mutant (orange). When 7 weeks old, Kitaake rice plants were inoculated with P. fuscovaginae by injecting a bacterial solution. Disease was evaluated at 2, 4 and 8 days post inoculation (DPI) (**a**). The levels of abscisic acid (**b**), jasmonate (**c**), auxin (**d**) and salicylic acid (**e**) were measured in sheath samples collected at 1, 2, 4 and 8 DPI. Black boxplots show the results of the healthy control. All boxplots represent the median with the first and third quartile, the whiskers show the minimum and maximum values. Boxplots marked different letters are significantly different (Wilcoxon rank sum test, *n* = 25, α = 0.05 (**a**) or ANOVA, Dunn’s or Mood’s Median test, *n* = 5, α = 0.05 (**b, c, d, e**))

The role of the lipopeptide fuscopeptin in these hormonal responses was investigated by inoculating the rice plants with *P. fuscovaginae* wild type strain UPB0736 and its fuscopeptin mutant delta445. Preliminary results (Fig. S1) showed a stronger response in the stem and lesions were larger on the third youngest leaf sheath so the latter was used for disease evaluation and sampled for hormone measurements. The results in Fig. 5c show a response of the rice plants to *P. fuscovaginae* at 2 DPI. Therefore, disease was scored at 2, 4 and 8 DPI. At every time point, inoculation with the wild type strain UPB0736 caused similar patterns of infection, while the fuscopeptin mutant strain delta445 was significantly less virulent (Fig. 6a).

To reduce the effect of the inoculation procedure, samples were collected at 1 DPI, 2 DPI, 4 DPI and at 8 DPI (Fig. 6). At the first three sampling points (1, 2, 4 DPI), ABA levels were similar in all conditions. By 8 DPI, plants inoculated with UPB0736 showed elevated ABA levels while plants inoculated with the fuscopeptin mutant delta445 did not show any ABA response (Fig. 6a). By 2 DPI, JA levels were slightly elevated in response to both *P. fuscovaginae* strains. Only in plants inoculated with UPB0736, JA levels stayed elevated during the rest of the infection process (Fig. 6b). For IAA, on the other hand, an early transient response to the wild type strain was observed. At 1 and 2 DPI, IAA levels in the wild type infected plants were more than doubled compared to the healthy control plants and plants inoculated with delta445 (Fig. 6c). By 8 DPI, SA levels were slightly elevated in UPB0736 infected plants but the levels did not significantly differ from the plants inoculated with delta445 (Fig. 6d).

### Yield Losses

The yield losses, as a result of sheath rot infection, were investigated in rice plants that were inoculated with isolates of *S. oryzae* and *P. fuscovaginae* that differed in virulence (Figs. 1 and 5a). Disease severity and various yield parameters were recorded at 6 and 8 weeks post inoculation (WPI) with respectively *S. oryzae* and *P. fuscovaginae*. At 6 WPI, the infection by the pathogenic *S. oryzae* strain, IBNG0008, had strongly advanced (average 842 ± 209 mm^2^). Also the infection by the less pathogenic isolate RFNG30 had further progressed (average 96 ± 53 mm^2^). According to the observed lesions on the rice sheath, *P. fuscovaginae* infection did not seem to have advanced much in 8 weeks (UPB0736, average 213 ± 133 mm^2^; delta445, average 11 ± 10 mm^2^). However, inside the stem, the rice plants showed necrosis at 8 WPI of both *P. fuscovaginae* strains but also some healthy control plants, injected with sterile saline solution, were necrotic inside.

**Fig. 7:**
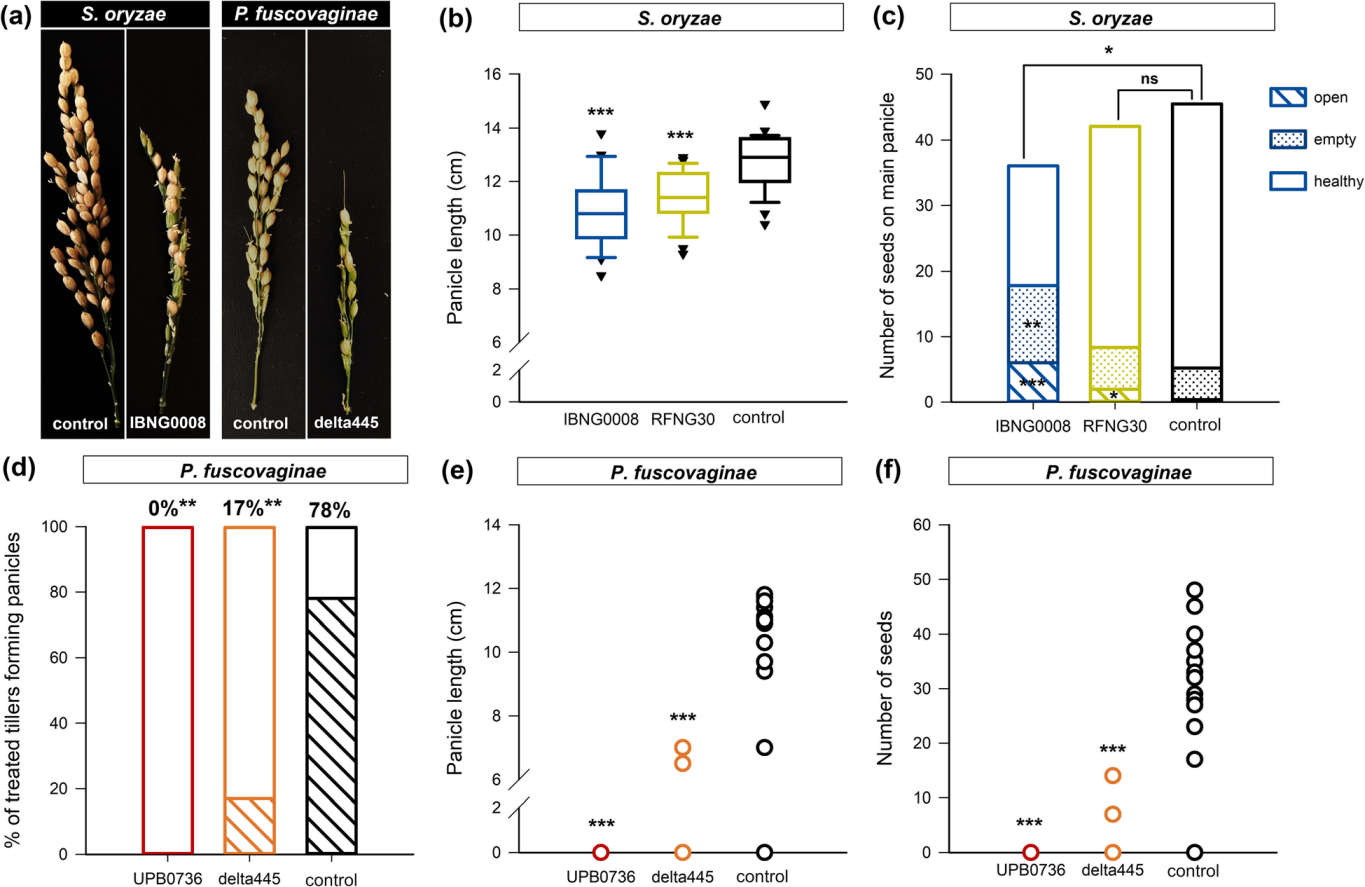
Grain yield parameters of Sarocladium oryzae and Pseudomonas fuscovaginae infected plants. The main tiller of 7 weeks old Kitaake rice plants was inoculated with *S. oryzae* or P. fuscovaginae. IBNG0008 is shown in dark blue, RFNG30 in light green and the healthy control, inoculated with a healthy rice grain, in black. P. fuscovaginae wild type strain UPB0736 is shown in red, delta445 in orange and as a healthy control, shown in black, a sterile saline solution was injected. (**a**) A fully ripened panicle, 6 or 8 weeks after inoculation with respectively *S. oryzae* and P. fuscovaginae. (**b**) Boxplots represent the length of the main panicle and (**c**) stacked bars show the average amount of open, empty and healthy seeds on the main panicle of plants inoculated with *S. oryzae*. Yield parameters of plants inoculated with P. fuscovaginae are represented by (**d**) stacked bars showing the proportion of the inoculated tillers forming a panicle and dots represent the length of the main panicle (**e**) or the amount of seeds produced by the main panicle of each sample (**f**). The boxplots show the median with the first and third quartile, the whiskers show the minimum and maximum values. Outliers and extreme values are represented by crosses. These data show the results of one experiment with 25 replicates (**b-c**) or 18 replicates (**d-f**). Conditions that differ significantly from healthy control plants are represented by asterisks (Mann Whitney or Mood’s Median, *n* = 18 or *n* = 25, α = 0.05 (**a-c, e-f**). Asterisks mark conditions that influence panicle formation significantly (Logistic binary regression, n = 18 (**d**))

Both sheath rot pathogens affected the yield (Fig. 7a). Plants infected with *S. oryzae* IBNG0008 produced shorter panicles with less seeds, compared to healthy plants. RFNG30 infection also reduced panicle length (Fig. 7b). The seeds produced by the plants infected with the IBNG0008 were significantly more open and more empty, compared to the control plants. Plants infected with the less pathogenic strain RFNG30 produced as much seeds as healthy plants, but the seeds were more often open (Fig. 7a,c). Plants inoculated with *P. fuscovaginae* wild type strain UPB0736 did not produce panicles at all (Fig. 7d,e). Inoculation with the fuscopeptin mutant delta445 also led to a significant decrease in panicle and seed production compared to the control plants (Fig. 7f).

## Discussion

In this research, the interaction of the rice sheath rot pathogens *S. oryzae* and *P. fuscovaginae* with their host was investigated. As both pathogens are toxin producers, we studied if their toxin production can be held responsible for the observed physiological changes, for their virulence and for the yield losses they cause. For this, isolates that differ in the *in planta* production of cerulenin, helvolic acid and fuscopeptin were used.

### ABA, JA and IAA Levels during *Sarocladium oryzae* Infection

This research revealed strong differences in the triggered host immune responses among the *S. oryzae* isolates. Early during infection, a stress response through JA accumulation was observed for all isolates. Only in plants infected by the most virulent isolates, JA levels stayed elevated during the rest of the infection. Simultaneously, ABA and IAA levels increased too while for the less virulent isolates, all phytohormone levels stayed unaltered from 2 DPI on. In previously published work, we showed that helvolic acid levels at 6 DPI are correlated with *S. oryzae* virulence (Fig. S2) (Peeters et al. 2020). In this study we measured high concentrations of ABA, JA and IAA in the lesions caused by these virulent helvolic acid producers both at 4 DPI and at 6 DPI (Fig. S3). However, the less virulent isolate RFNG41 produced similar levels of helvolic acid but it did not trigger strong hormonal responses. This was also observed for the isolates BDNG0005 and CBS180.74 at 6 DPI which indicates that next to helvolic acid, another compound is needed for virulence. Based on our results, cerulenin does not seem to be the missing virulence factor as isolate BDNG0025 caused severe sheath rot symptoms but produced only trace amounts of cerulenin. Moreover, the weakly pathogenic isolate RFRG2 produces high levels of cerulenin but no helvolic acid and BDNG0005 produces significant levels of both toxins but was the least virulent isolate. We showed before that *S. oryzae* strains segregate in three phylogenetic groups and that two of these groups (group 1 and 2) show sectorization, a process that affects helvolic acid production (Peeters et al. 2020). All mentioned weakly pathogenic isolates belong to these instable groups. These groups have recently been reclassified as *Sarocladium oryzae* (corresponding to our group 1) and *Sarocladium attenuatum* (corresponding to our group 2) (Ou et al. 2020; Table 1). The three isolates that caused severe sheath rot symptoms and triggered strong IAA, ABA and JA responses, on the other hand, belong to the third phylogenic group. This group was shown to be more stable (Peeters et al. 2020) and has recently been proposed as a separate species, called *Sarocladium sparsum* (Ou et al. 2020; Table 1). The fact that ABA-, JA- and IAA-accumulation was only observed in case of severe infection and that their levels positively correlate with the lesion area (Fig. S2), indicates that these phytohormone responses are no sign of disease resistance. They are rather susceptibility factors acting in favour of *S. oryzae*.

### ABA, JA and IAA Levels during *Pseudomonas fuscovaginae* Infection

When plants were inoculated with *P. fuscovaginae* by injecting a bacterial solution, all conditions showed a strong increase of ABA that was probably triggered by the wounding (Pieterse et al. 2012). As mock-treated plants showed the strongest response, both *P. fuscovaginae* wild type strains seem to be able to partially suppress this ABA stress response. Later during infection, ABA and JA levels increased and reached by 8 DPI similar levels as observed already at 4 DPI during *S. oryzae* infection. While the production profile of JA was rather variable, ABA accumulation was clearly delayed compared to *S. oryzae* infection. Symptom development, however, started earlier, indicating that the postponement of the ABA response could be a possible virulence strategy of *P. fuscovaginae*. Because of its role in pathogen-induced stomatal closure, ABA is often targeted by pathogen effectors to facilitate host penetration. For example, the *P. syringae* toxin coronatine forces stomatal opening by inhibiting ABA signalling (Hok et al. 2010). Moreover, our results show that in experiments where ABA reached higher levels, *P. fuscovaginae* caused less symptoms. Although ABA is predominantly known as a repressor of plant immunity, it has been reported to induce resistance against *Cochliobolus miyabeanus* in rice (De Vleesschauwer et al. 2010, 2013). The role of JA is less clear. In case of severe infection, we observed an earlier JA accumulation and the JA levels at 8 DPI reached in both experiments similar levels, regardless the size of the lesions. While both sheath rot pathogens triggered JA and ABA accumulation, IAA levels showed a different pattern. Already early during infection, *P. fuscovaginae* caused a short, transient accumulation of IAA. During more severe infections, the accumulation of IAA started earlier and reached higher levels. This indicates that IAA possibly enhances the infection and acts as a sensitivity factor. Auxin is known to promote susceptibility to various rice pathogens including *Xanthomonas oryzae*, *Pyricularia oryzae* and to the syringopeptin producer, *P. syringae* (Kazan and Lyons 2014). These pathogens promote auxin production by the plant and secrete auxin themselves, using it as a virulence factor (De Vleesschauwer et al. 2013). In the genome of *P. fuscovaginae*, a possible gene that encodes for tryptophan 2-monooxygenase, an enzyme of the auxin anabolism, was found (Quibod et al. 2015). The measured IAA could thus be at least partially of bacterial origin. The use of a fuscopeptin mutant confirmed that the observed phytohormone responses are correlated with the symptom development as this strain caused significantly smaller necrotic lesions (Patel et al. 2014; Weeraratne et al. 2020). Moreover, ABA nor JA accumulated in plants that were inoculated with the fuscopeptin mutant and no transient IAA peak was observed.

### The Interaction of Rice with the Sheath Rot Pathogens and their Toxins

Except for small variations in the SA levels, no clear SA response was observed towards sheath rot infection. The basal SA levels in the healthy control tissue varied between the pathogens. For *S. oryzae*, the rice plants were inoculated in the axil of the second youngest leaf and the sheath of this leaf was sampled. *P. fuscovaginae*, on the contrary, was injected into the lower parts of the plants. Consequently, we sampled other sheath layers from the lower plant part, which could explain why lower SA levels were measured in the *P. fuscovaginae* samples (Silverman et al. 1995).

Both JA and ABA accumulated during sheath rot infection but ABA seemed to adversely affect disease resistance. The timing and pattern of IAA accumulation, on the other hand, differed between the sheath rot pathogens. Together with JA, IAA seems to act as a susceptibility factor in sheath rot, regardless the causal agent. The overlap in the mode of action of helvolic acid and fuscopeptin could possibly explain why the sheath rot pathogens trigger partially similar disease responses and cause almost identical symptoms. *F. verticillioides* and *Cercospora beticola* are also known to block H^+^-ATPases (Elmore and Coaker 2011). *F. verticillioides* produces the amphiphilic toxin fumonisin B1 (FB1) which affects membrane integrity and is produced by various *Fusarium spp.* including sheath rot pathogens (Abbas et al. 1999; Kushiro et al. 2012; Xing et al. 2013; Asai and Shirasu 2015). The toxin beticolin, produced by *C. beticola*, is a Mg^2+^ scavenger like helvolic acid (Gom et al. 1996). Interestingly, plants also accumulate ABA and JA in response to *F. verticillioides* or *C. beticola* (Schmidt et al. 2008; Lanubile et al. 2014; Vaughan et al. 2014, 2016). The synergistic action of ABA and JA is well described for the abiotic stress response and in the defence against insect herbivory but has not often been observed in plant disease responses (De Vleesschauwer et al. 2014; Nguyen et al. 2016). JA and ABA stimulate each other’s biosynthesis and, in *Arabidopsis thaliana*, their signalling pathways act synergistically through MYC2. The TF MYC2 is a master regulator of the JA/ABA defence response against pests and abiotic stress (Kazan and Manners 2013; Nguyen et al. 2016). Moreover, MYC2 antagonizes the ERF-dependent JA/ET defence response against necrotrophs (Pieterse et al. 2012; Nguyen et al. 2016). Resistance to *C. beticola* in sugar beet and to *F. verticillioides* in maize has been attributed to the JA/ET signalling pathway while the role of ABA is not well understood for these pathosystems (Schmidt et al. 2008; Lanubile et al. 2014; Vaughan et al. 2014, 2016). Moreover, overexpression of WRKY13 in rice generates partial resistance against *S. oryzae* (Lilly and Subramanian 2019) which, in high concentrations, blocks the JA-pathway and the abiotic stress response (Qiu et al. 2007; Xiao et al. 2013; De Vleesschauwer et al. 2014).

For both sheath rot pathogens, the molecular mechanisms underlying the infection process remain to be elucidated and further research is needed to clarify the role of the phytohormones. Our results suggest that ERF-dependent JA/ET pathway possibly regulates the defence response against *S. oryzae* while the ABA/JA branch may provide resistance against *P. fuscovaginae* (Pieterse et al. 2012). It would be interesting to measure ET during infection and to verify the effect of elevated ET levels on the disease development of these pathogens. Moreover, the origin of the observed IAA accumulation during *P. fuscovaginae* infection should be studied and also which extra virulence factors distinguish *S. sparsum* from the less virulent groups.

### Yield Losses Caused by Sheath Rot Infection

Next to necrotic lesions on the sheath, *S. oryzae* and *P. fuscovaginae* are known to cause significant yield losses (Sakthivel 2001; Razak et al. 2009; Panda and Mishra 2019). This study showed that the most virulent, toxin-producing isolates strongly affected seed production. The could possibly be attributed to the blockage of the H^+^-ATPases by the toxins helvolic acid and fuscopeptin (Gachon et al. 1973; Yoshimura 1978; Batoko et al. 1998). These enzymes play indispensable roles in phloem loading (Falhof et al. 2016) and photosynthesis (Feng et al. 2016) which are important processes to maintain a proper source/sink relationship (Kim et al. 2009a, 2009b; Tamaki et al. 2015). Grain filling strongly depends on the transport of non-structural carbohydrates (NSC) towards sink organs through the phloem (Tamaki et al. 2015; Yu et al. 2015). Blockage of proton pumps prevents the export of assimilates to sink organs (Elmore and Coaker 2011). Grain production is also directly affected by necrosis in the leaf sheath. This causes a decrease of the carbon reserves in the rice straw (Wang et al. 2017; Zhang et al. 2019). In rice, spikelet development and grain filling are regulated by ABA, JA, IAA and ET (Kim et al. 2009a, 2009b; Javid et al. 2011; Tamaki et al. 2015). There is a positive crosstalk between JA and ABA biosynthesis and signalling in the suppression of grain production, often with regard to abiotic stresses such as drought (Yang 2001; Travaglia et al. 2007; Kim et al. 2009b). Kim et al. (2009a, 2009b) reports both phytohormones affecting the same grain yield parameters as observed in this study. Additionally, we showed that *S. oryzae* infected plants produce significantly more open grains. Altered JA levels are known to result in empty seeds with open glumes (Li et al. 2018). None of the plants inoculated with the *P. fuscovaginae* wild type strain produced panicles and, of the plants inoculated with the fuscopeptin mutant, only 17% did. This mutant still produces syringotoxin, which affects photosynthesis and phloem loading too (Mott and Takemoto 1989; Batoko et al. 1998).

## Conclusions

While both *S. oryzae* and *P. fuscovaginae* continue spreading and causing significant yield losses, the need for effective control measures is high. For this, it is of crucial importance to understand the mode of action of the sheath rot pathogens. With this research, we provide information on the host immune response and possible targeted hormonal pathways of sheath rot and sheath brown rot, paving the way to controlling these diseases. This study shows that helvolic acid and fuscopeptin production strongly relate to the hormonal response towards respectively *S. oryzae* and *P. fuscovaginae* and to their virulence. ABA and JA are positively correlated with *S. oryzae* virulence which suggests that the synergistic action of JA/ET could possibly provide resistance against *S. oryzae*. As ABA seems to act as a resistance factor against *P. fuscovaginae*, our results suggest that the JA/ET branch could possibly provide susceptibility. This obviously needs further investigation. Furthermore, our research stresses the importance of taking variability of the pathogen population into account when investigating virulence strategies and control measures.

## Material and Methods

### Plant Materials & Growth Conditions

All experiments were performed on 7 week-old plants of the *japonica* type rice (*Oryza sativa*) cv. Kitaake. Seed germination and growth conditions were as described in Peeters et al. (2020). Briefly, germinated seedling were planted after 7 days in sterile potting soil (Structural; Snebbout, Kaprijke, Belgium) in perforated plastic trays (22 × 15 × 6 cm). Each tray contained six plants and rice plants were grown for 6 weeks in a greenhouse at 28 °C at 60% relative humidity (RH). The plants were watered 6 times a day with a flooding system and were weekly supplemented with 0.2% iron sulphate and 0.1% ammonium sulphate.

### Fungal Inoculation and Disease Rating

Table 1 lists the *Sarocladium* isolates that were used in this study. Their morphology, toxin production and virulence on Kitaake rice plants have been described before (Peeters et al. 2020). Rice plants were inoculated using the standard grain inoculum technique, following Sakthivel and Gnanamanickam (1987). The preparation of the inoculum, the inoculation procedure and the conditions during incubation are described in detail by Peeters et al. (2020). Briefly, rice grains were autoclaved twice after soaking them in water for 60 min. For each 4 g of rice grains, one plug (diameter = 5 mm) from the edge of a 14-day-old fungal colony was added together with 1 mL of sterile distilled water. After 14 days of incubation at 28 °C, one fully colonized grain was introduced in the junction point between the sheath of the second youngest plant leaf and the stem. To maintain humidity, inoculation points were covered with moist cotton and wrapped with Parafilm. Plants were incubated under growth chamber conditions (28 °C day/28 °C night, 12/12 light regimen, and 85% relative humidity during the first 24 h, 65% relative humidity during day 2–8). For each treatment, 20 plants (4 trays × 5 plants) or 25 plants (5 trays × 5 plants) were used and disease was evaluated after respectively 4 or 6 days of incubation by measuring the lesion area (mm^2^) (Peeters et al. 2020).

### Bacterial Inoculation and Disease Rating

For the experiments with *P. fuscovaginae*, two wild type strain were used. The Australian isolate MB194 (a.k.a. DAR777800) and the wild type strain UPB0736 from Madagascar and its syringopeptin synthetase mutant delta445 were provided by Vittorio Venturi (Patel et al. 2014). To prepare bacterial inoculum, isolates were taken from the − 80 °C collection, plated on King’s Medium B (KB) and incubated for 48 h at 28 °C. Following, one single bacterial colony was transferred to Luria-Bertani broth (LB) and shaken (150 rpm) for 20 h at 28 °C. After centrifuging the bacterial liquid culture (13,000 rpm, 2 min), the supernatant was discarded. The pellet, containing the bacteria, was dissolved in sterile saline solution (0.85% NaCl) and diluted to an optical density of 0.1 at 620 nm. Bacteria were inoculated by injecting a bacterial suspension in the main tiller of the plant. For this, a volume of 0.5–1 ml was injected with a 1 ml syringe at 10 cm above the soil surface until droplets formed in the axil of the youngest leaf (Rott et al. 1991). Control plants were injected with a sterile saline solution. First, the plants were incubated for 24 h at 85% relative humidity (RH) (28 °C, 12/12 light/dark). During the remaining incubation period, the RH was lowered to 65%. For each treatment, 25 plants (5 trays × 5 plants) were used and disease was evaluated by measuring the lesion area after 2, 4 and 8 days of incubation (Peeters et al. 2020).

### Toxin and Phytohormone Extraction and Quantification

At different time points during the pathogenicity tests with *S. oryzae* and *P. fuscovaginae*, described above, samples were collected for phytohormone and toxin analysis. For this, 8 cm pieces of the sheath of the main panicle, containing the inoculated area, were collected. Of each tray, five plants were pooled in one sample, immediately immersed in liquid nitrogen and stored at − 80 °C until further processed. Extraction and quantification of the toxins cerulenin and helvolic acid from the rice sheath was performed as described before (Peeters et al. 2020). The phytohormones ABA, JA, SA and IAA were extracted during the same extraction procedure as the toxins. The analytical method has been validated by Haeck et al. 2018. Briefly, before extraction, samples were ground using a tissue lyser and 100 mg of fine powder was extracted with cold modified Bieleski solvent (methanol, ultrapure water and formic acid, 75:20:5, v:v:v). Afterwards, supernatant was filtered (30 kDa Amicon Ultra centrifugal filter, Merck Millipore, Overijse, Belgium) and the resulting filtrate was reduced to dryness under N_2_ at 20 °C (Turbovap® evaporator). After dissolving the dry filtrate with reconstitution solvent (methanol/water (20:80 v/v) with 0.1% formic acid), instrumental analysis was immediately performed. For this, an ultra-high performance liquid chromatography system, coupled to a quadrupole-orbitrap mass spectrometer was used. Chromatographic separation was achieved on a Accela U-HPLC system (Thermo Fisher Scientific, Erembodegem, Belgium), coupled to an Accela Autosampler and Degasser and equipped with a Nucleodur C18 column (50 × 2 mm; 1.8 μm d_p_, Macherey-Nagel, Düren, Germany). Mass spectrometric analysis was carried out using a Q-Exactive™ bench top HRMS (Thermo Fisher Scientific), equiped with a heated electrospray ionization source. The positive ionization mode was used for the measurement of IAA, helvolic acid and cerulenin; the negative ionisation mode for the measurement of ABA, JA and SA (Table S2). The measurements were performed in targeted single ion monitoring (t-SIM) at a mass resolving power of 70,000 full width at half maximum (FWHM).

### Grain Yield Components Quantification

When 7 weeks old, rice plants were inoculated with *S. oryzae* or *P. fuscovaginae* as described above. For *S. oryzae*, 25 plants (5 trays × 5 plants) were used per treatment and for *P. fuscovaginae*, 18 plants (3 trays × 6 plants) were used per treatment. The plants were incubated in growth chambers (65% RH, 28 °C, 12/12 light/dark) for 6–8 weeks. When the main tillers of the healthy control plants had a yellow flag leaf and the last seed was fully filled and ripened, panicles were harvested. For each panicle, the length was measured and the open, empty and healthy seeds were counted.

### Statistical Analysis

The statistical analyses were performed in R-4.0.0 (R Core Team 2020) and SPSS 26.0 (IBM SPSS, Armonk, NY, USA). Pairwise and multiple comparison analysis were performed with the packages lme4 v1.1–23 (Bates et al. 2015), car-3.0-7 (Fox and Weisberg 2019), afex-0.27-2 (Singmann et al. 2020), emmeans-1.4.6 (Lenth 2020), dunn.test-1.3.5 (Dinno 2017). All statistic tests were performed with a fixed significance level of 0.05.

## Supplementary Information

**Additional file 1.**

## Data Availability

The datasets used and/or analysed during the current study are available from the corresponding author on reasonable request.

## Abbreviations

ABA: Abscisic acid
AUX: Auxin
DPI: Days post inoculation
ET: Ethylene
FWHM: Full width at half maximum
IAA: Indole acetic acid
JA: Jasmonate
KB: King’s Medium B
LN: Luria-Bertani broth
PM: Plasma membrane
RH: Relative humidity
SA: Salicylic acid
TF: Transcription factor
t-SIM: Targeted single ion monitoring
WPI: Weeks post inoculation
