# Supplementary material for: Auxin, Abscisic Acid and Jasmonate Are the Central Players in Rice Sheath Rot Caused by Sarocladium oryzae and Pseudomonas fuscovaginae
Source: Rice (N Y). 2020 Nov 26;13:78. doi: 10.1186/s12284-020-00438-9 (PMC7691414; doi:10.1186/s12284-020-00438-9)
Supplement: Supplementary file 1 — Additional file 1. [file 12284_2020_438_MOESM1_ESM.docx]

# **Supporting information - Auxin, abscisic acid and jasmonate are the central players in rice sheath rot caused by Sarocladium oryzae and Pseudomonas fuscovaginae**

**Peeters, K. J.., Ameye, M., Demeestere, K., Audenaert, K., Höfte, M.**

**Table S1** Virulence data and the average cerulenin and helvolic acid production in rice sheaths at 6 days post inoculation of *S. oryzae* isolates (Peeters et al. 2020).

| ***S. oryzae* isolate** | **Cerulenin in planta**  **(ng g^-1^)** | **Helvolic acid in planta (ng g^-1^)** | **Lesion area on the sheath (mm²)** |
| --- | --- | --- | --- |
| IBNG 0008 | 58 | 5306 | 236 |
| IBNG 0009 | 258 | 7680 | 323 |
| BDNG 0025 | 5 | 3892 | 227 |
| RFRG 2 | 1196 | 0 | 60 |
| CBS 180.74 | 338 | 729 | 30 |
| RFNG 30 | 0 | 2 | 38 |
| RFNG 122 | 1 | 3 | 41 |
| RFBG 3 | 0 | 0 | 46 |
| RFNG 41 | 1 | 461 | 50 |
| BDNG 0005 | 149 | 3193 | 21 |

**Table S2** The mass-to-charge ratio (m/z) of the precursor ions, their ionization mode and the retention time of the compounds measured with LC-MS/HRMS in this study.

| Compound | Ionization mode | Precursor ion (m/z) | Retention Time (min) |
| --- | --- | --- | --- |
| Indole-3-acetic acid (IAA) | + | 176.0706 | 4.15 |
| d-IAA | + | 181.1020 | 4.15 |
| Salicylic acid (SA) | - | 137.0244 | 4.80 |
| d-SA | - | 141.0495 | 4.80 |
| Abscisic acid (ABA) | - | 263.1289 | 4.98 |
| d-ABA | - | 269.1665 | 4.98 |
| Jasmonate | - | 209.1183 | 5.62 |
| Cerulenin | + | 246.1101 | 5.64 |
| Helvolic acid | + | 591.2928 | 8.15 |


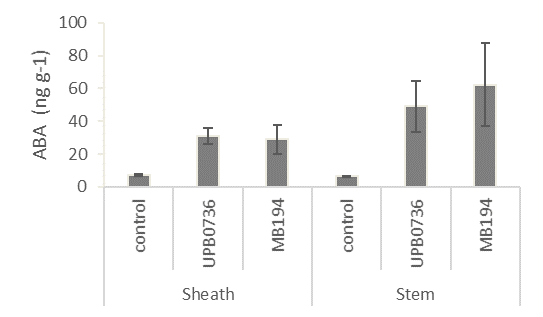

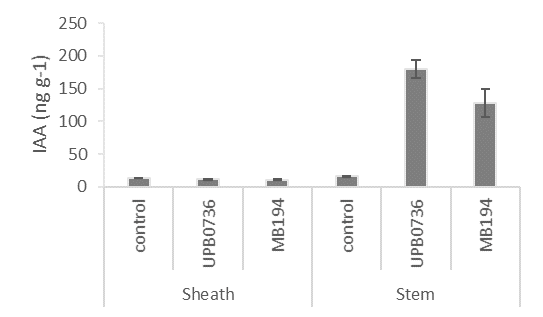

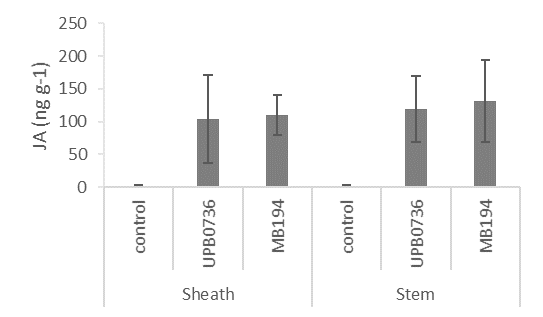

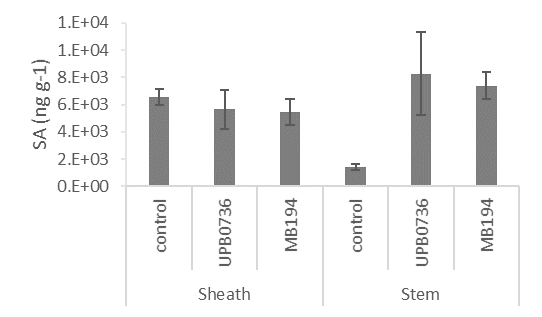


**Figure S1**. Levels of abscisic acid (**a**), jasmonate (**b**) auxin (**c**) and salicylic acid (**d**) in the rice sheath during infection of Pseudomonas fuscovaginae wild type strains. When 7 weeks old, rice plants were inoculated with P. fuscovaginae by injecting a bacterial solution and samples of the rice sheath were collected at 8 days post inoculation.

**(a)**

**(b)**

**(c)**

**(d)**


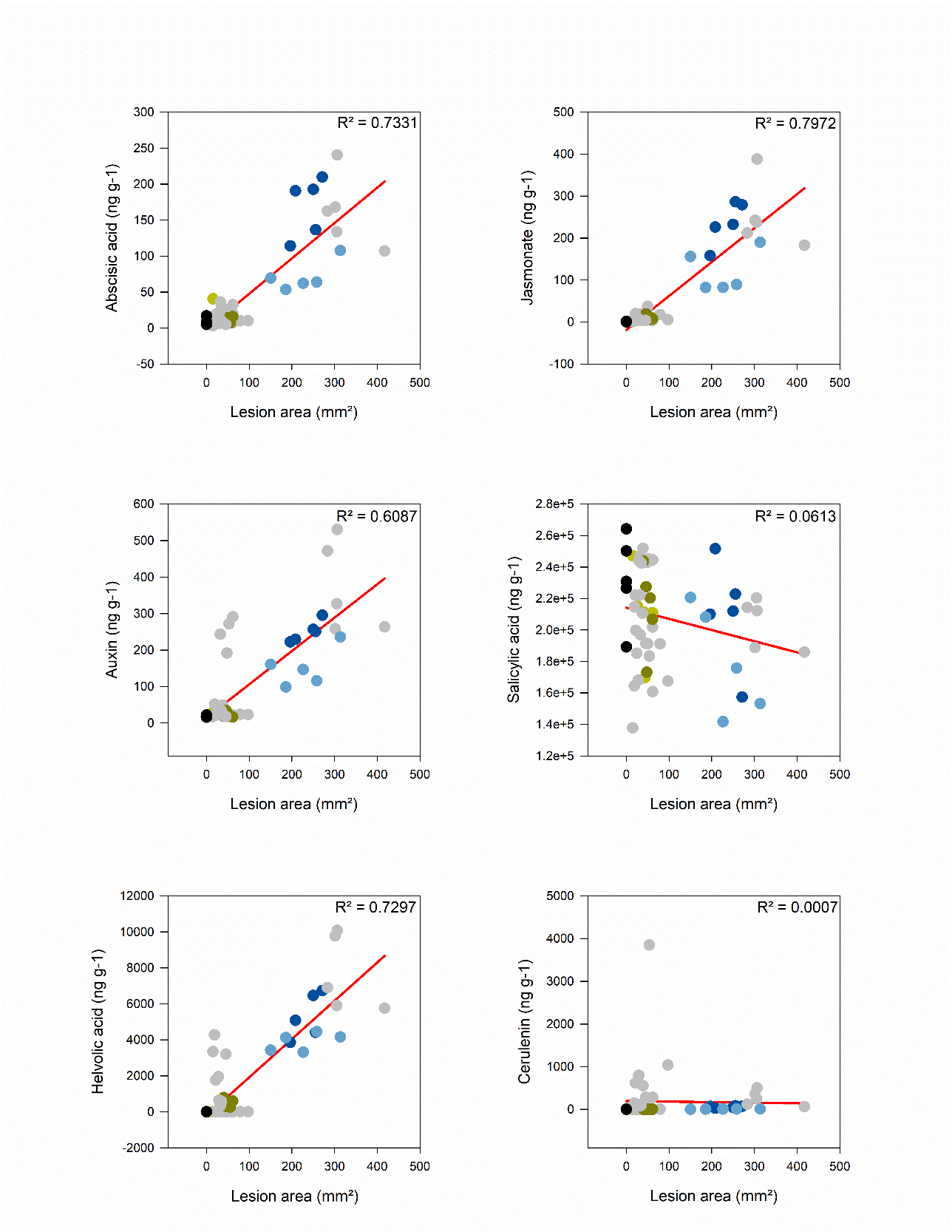


**Figure S2.** Associations between the lesion size (mm²) (Peeters et al. 2020) and the measured levels of the phytohormones abscisic acid (**a**), jasmonate (**b**), auxin (**c**), salicylic acid (**d**) and the toxins helvolic acid (**e**) and cerulenin (**f**) (Peeters et al. 2020). When 7 weeks old, rice plants were inoculated with *S. oryzae* using the standard grain inoculum technique. At 6 DPI, disease was scored by measuring the lesion area (mm²). Next, sheath samples were collected and analyzed with liquid chromatography high-resolution mass spectrometry. Scatterplots show the associations and the linear correlation coefficients. The colors correspond to the colors used in the manuscript with *S. oryzae* isolate IBNG0008 represented by dark blue dots, BDNG0025 by light blue dots, RFNG41 by dark green dots and RFNG30 by light green dots. Black dots represent the healthy control plants.

**(a) (b)**

**(c) (d)**

**(e) (f)**


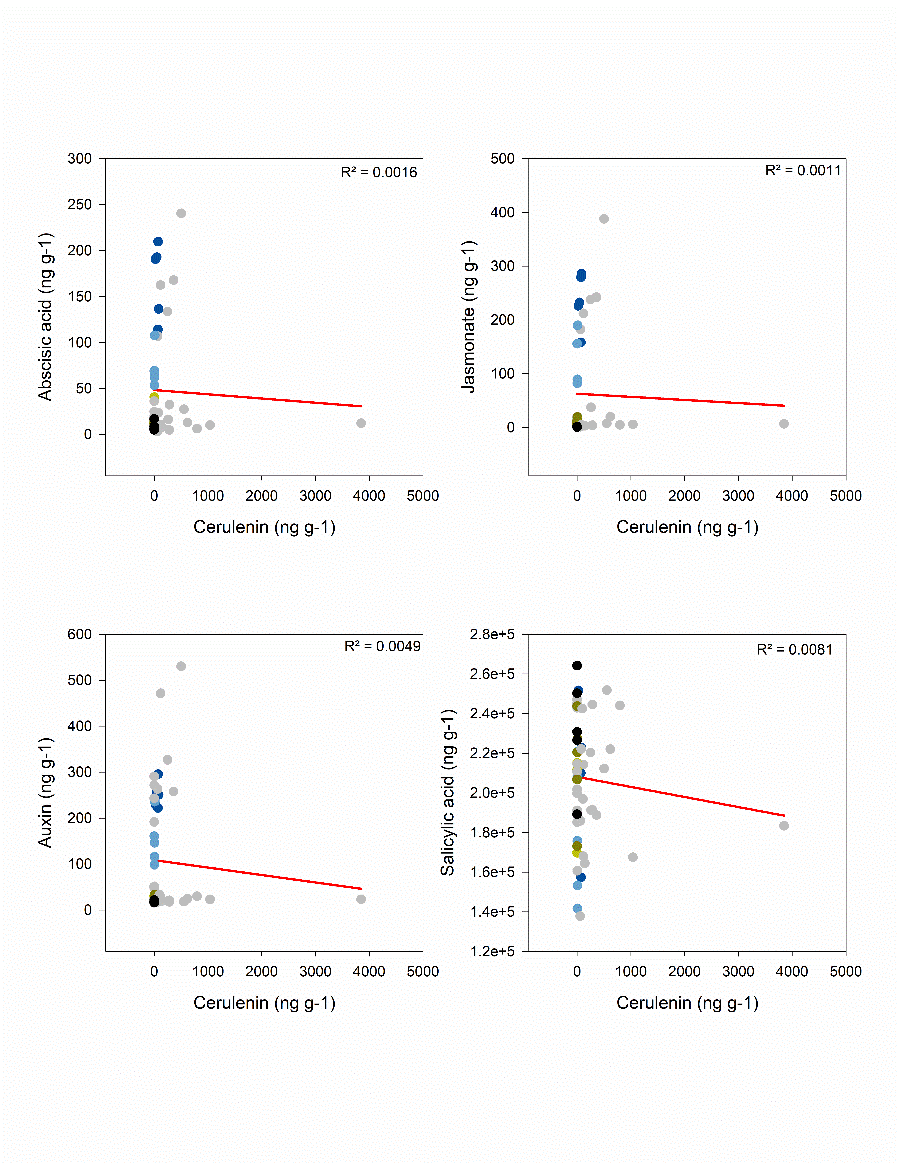

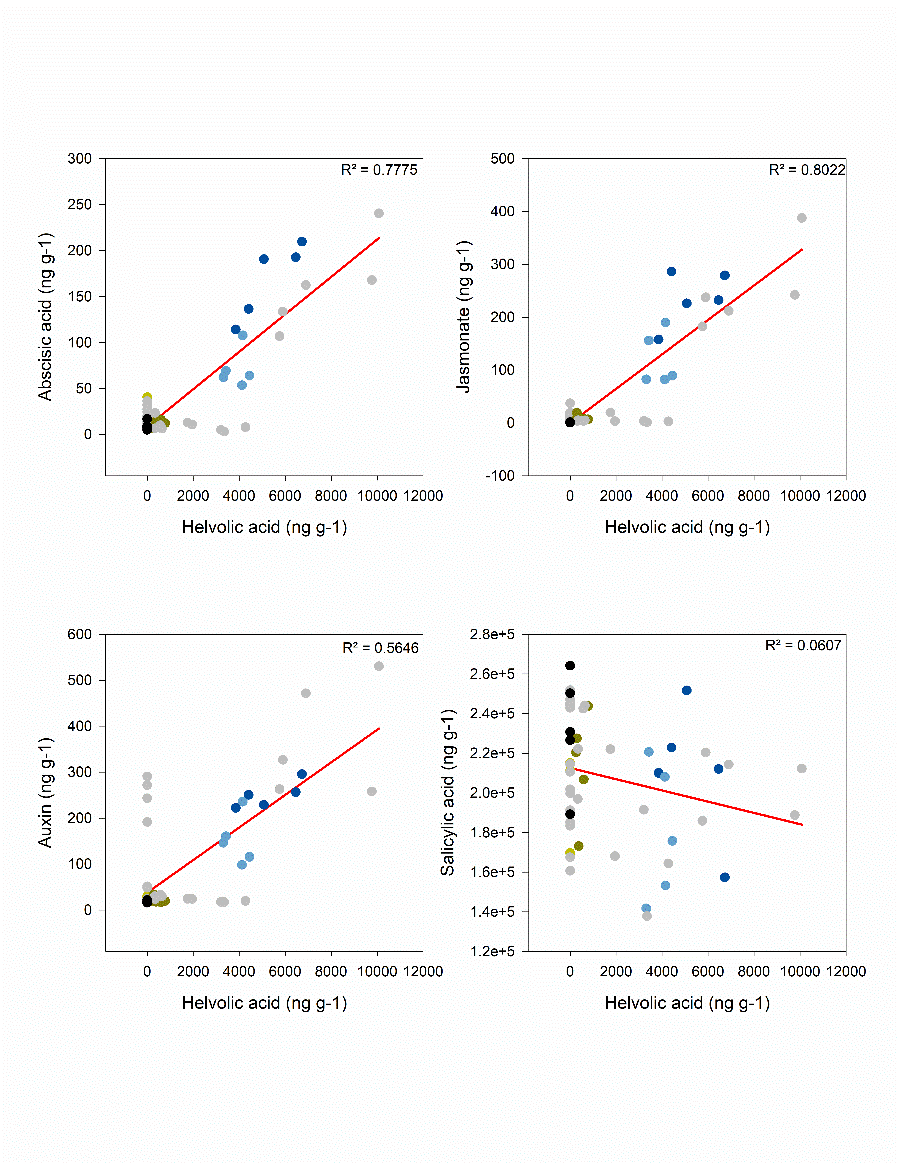


**Figure S3**. Associations between cerulenin (**a-d**) and helvolic acid (**e-h**) production (Peeters et al. 2020) in the rice sheath and the measured levels of the phytohormones abscisic acid (**a,e**), jasmonate (**b,f**), auxin (**c,g**) and salicylic acid (**d,h**). When 7 weeks old, rice plants were inoculated with *S. oryzae* using the standard grain inoculum technique. At 6 DPI, disease was scored by measuring the lesion area (mm²). Next, sheath samples were collected and analyzed with liquid chromatography high-resolution mass spectrometry. Scatterplots show the associations and the linear correlation coefficients. The colors correspond to the colors used in the manuscript with *S. oryzae* isolate IBNG0008 represented by dark blue dots, BDNG0025 by light blue dots, RFNG41 by dark green dots and RFNG30 by light green dots. Black dots represent the healthy control plants.

**(a) (b)**

**(c) (d)**

**(e) (f)**

**(g) (h)**
